# Comparison of artificial intelligence and human-based prediction and stratification of the risk of long-term kidney allograft failure

**DOI:** 10.1038/s43856-022-00201-9

**Published:** 2022-11-23

**Authors:** Gillian Divard, Marc Raynaud, Vasishta S. Tatapudi, Basmah Abdalla, Elodie Bailly, Maureen Assayag, Yannick Binois, Raphael Cohen, Huanxi Zhang, Camillo Ulloa, Kamila Linhares, Helio S. Tedesco, Christophe Legendre, Xavier Jouven, Robert A. Montgomery, Carmen Lefaucheur, Olivier Aubert, Alexandre Loupy

**Affiliations:** 1 Université de Paris Cité, INSERM U970, PARCC, Paris Translational Research Centre for Organ Transplantation, Paris, France; 2 Kidney Transplant Department, Saint-Louis Hospital, Assistance Publique - Hôpitaux de Paris, Paris, France; 3 NYU Langone Transplant Institute, NYU Langone Health, New York, NY USA; 4 Department of Medicine, Division of Nephrology, David Geffen School of Medicine at UCLA, Los Angeles, CA USA; 5 Department of Surgery, Thomas E. Starzl Transplantation Institute, University of Pittsburgh, Medical Center, Pittsburgh, PA USA; 6 Kidney Transplant Department, Bicêtre Hospital, Assistance Publique – Hôpitaux de Paris, Kremlin-Bicêtre, France; 7 Medical Intensive Care Unit, Saint-Louis Hospital, Assistance Publique - Hôpitaux de Paris, Paris, France; 8 Department of Physiology, Assistance Publique-Hôpitaux de Paris, Hôpital Européen Georges Pompidou, Paris, France; 9 The First Affiliated Hospital of Sun Yat-Sen University, Guangzhou, China; 10 Clinica Alemana de Santiago, Santiago, Chile; 11 Universidade Federal de Sao Paulo, Hospital do Rim, Escola Paulista de Medicina, Sao Paulo, Brazil; 12 Kidney Transplant Department, Necker Hospital, Assistance Publique-Hôpitaux de Paris, Paris, France; 13 Cardiology and Heart Transplant department, Pompidou hospital, Assistance Publique - Hôpitaux de Paris, Paris, France

**Keywords:** Kidney, Predictive markers, End-stage renal disease

## Abstract

**Background:**

Clinical decisions are mainly driven by the ability of physicians to apply risk stratification to patients. However, this task is difficult as it requires complex integration of numerous parameters and is impacted by patient heterogeneity. We sought to evaluate the ability of transplant physicians to predict the risk of long-term allograft failure and compare them to a validated artificial intelligence (AI) prediction algorithm.

**Methods:**

We randomly selected 400 kidney transplant recipients from a qualified dataset of 4000 patients. For each patient, 44 features routinely collected during the first-year post-transplant were compiled in an electronic health record (EHR). We enrolled 9 transplant physicians at various career stages. At 1-year post-transplant, they blindly predicted the long-term graft survival with probabilities for each patient. Their predictions were compared with those of a validated prediction system (iBox). We assessed the determinants of each physician’s prediction using a random forest survival model.

**Results:**

Among the 400 patients included, 84 graft failures occurred at 7 years post-evaluation. The iBox system demonstrates the best predictive performance with a discrimination of 0.79 and a median calibration error of 5.79%, while physicians tend to overestimate the risk of graft failure. Physicians’ risk predictions show wide heterogeneity with a moderate intraclass correlation of 0.58. The determinants of physicians’ prediction are disparate, with poor agreement regardless of their clinical experience.

**Conclusions:**

This study shows the overall limited performance and consistency of physicians to predict the risk of long-term graft failure, demonstrated by the superior performances of the iBox. This study supports the use of a companion tool to help physicians in their prognostic judgement and decision-making in clinical care.

## Plain language summary

The ability to predict the risk of a particular event is key to clinical decision-making, for example when predicting the risk of a poor outcome to help decide which patients should receive an organ transplant. Computer-based systems may help to improve risk prediction, particularly with the increasing volume and complexity of patient data available to clinicians. Here, we compare predictions of the risk of long-term kidney transplant failure made by clinicians with those made by our computer-based system (the iBox system). We observe that clinicians’ overall performance in predicting individual long-term outcomes is limited compared to the iBox system, and demonstrate wide variability in clinicians’ predictions, regardless of level of experience. Our findings support the use of the iBox system in the clinic to help clinicians predict outcomes and make decisions surrounding kidney transplants.

## Abstract (web summary)

Divard, Raynaud et al. compare artificial intelligence (AI)-based predictions of kidney allograft failure based on electronic health records with those made by transplant physicians of varying levels of experience. The ability of physicians to predict allograft failure is limited, with superior performance seen for the AI system.

## Introduction

End-stage kidney disease (ESKD) represents a major global health burden with a prevalence exceeding 7 million^1^. Kidney transplantation is the best treatment for ESKD^2^ and is the most performed solid organ transplant in the world with more than 1,500,000 people living with a transplanted kidney according to the World Health Organization^1,3^. While short-term allograft survival has increased, improving long-term survival remains challenging^4^. The main limitations are the lack of new therapeutics as well as the absence of prediction systems that enable accurate risk stratification. Individual scoring systems that can risk stratify patients, serving as a companion tool for physicians, could improve patient management and facilitate the application of personalized medicine^4^.

However, accurately predicting patient outcomes can be challenging as physicians have to take into account numerous parameters from various sources, such as donor and recipient baseline characteristics^5,6^, follow-up parameters comprising immunological profile^7–9^, biomarkers, biopsy allograft phenotypes^10^, kidney function assessments^11–13^, treatment^14^, and parameters related to infections^15^, cancer^16^, and cardiovascular disease^17^. Consequently, accurately stratifying patient risk remains a difficult task for physicians^9^ and can lead to invasive examinations such as allograft biopsies or treatments with undesirable effects without any benefit for the patient.

For these reasons, several allograft failure prediction models have been developed^18–21^ to assist physicians in decision-making. The recently published iBox system is the most accurate and validated predictive algorithm in kidney transplantation to date and is currently undergoing review for regulatory endorsement by health authorities^9^. By adopting an integrative strategy using artificial intelligence to capture dependencies between numerous predictive factors, the iBox offers highly accurate allograft failure prediction performances and it has been validated in many centers, in distinct populations of transplant recipients, and in various clinical scenarios encountered in routine practice.

However, although existing risk prediction systems, including the iBox, have shown good predictive performances, none have been shown to outperform physicians. Before integrating these tools into clinical practice, it is necessary to investigate whether the predictions are more accurate than those made by physicians, which would provide a new perspective on the patient, potentially adjusting the prognostic judgment and therapeutic decisions.

Therefore, in this study, we aim to evaluate the ability of transplant physicians to predict and stratify the risk of long-term allograft failure and compare them to a validated artificial intelligence (AI) prediction algorithm. Transplant physicians were enrolled at distinct career stages, to assess their ability and consistency to predict and stratify long-term allograft failure, compared to the AI algorithm (the iBox system) based on the anonymized electronic health record of four-hundred kidney transplant recipients at one-year post-transplantation. Physicians’ predictions agreement and features that have led to their predictions were also investigated considering clinical experience. We show the overall limited performances of physicians to accurately predict individual long-term outcomes compared to the iBox system and demonstrate their wide heterogeneity of prognostic judgment, regardless of clinical experience. Taken together, we suggest the use of the iBox system to help physicians in the decision-making process.

## Methods

### Study design and participants

**Fig. 1:**
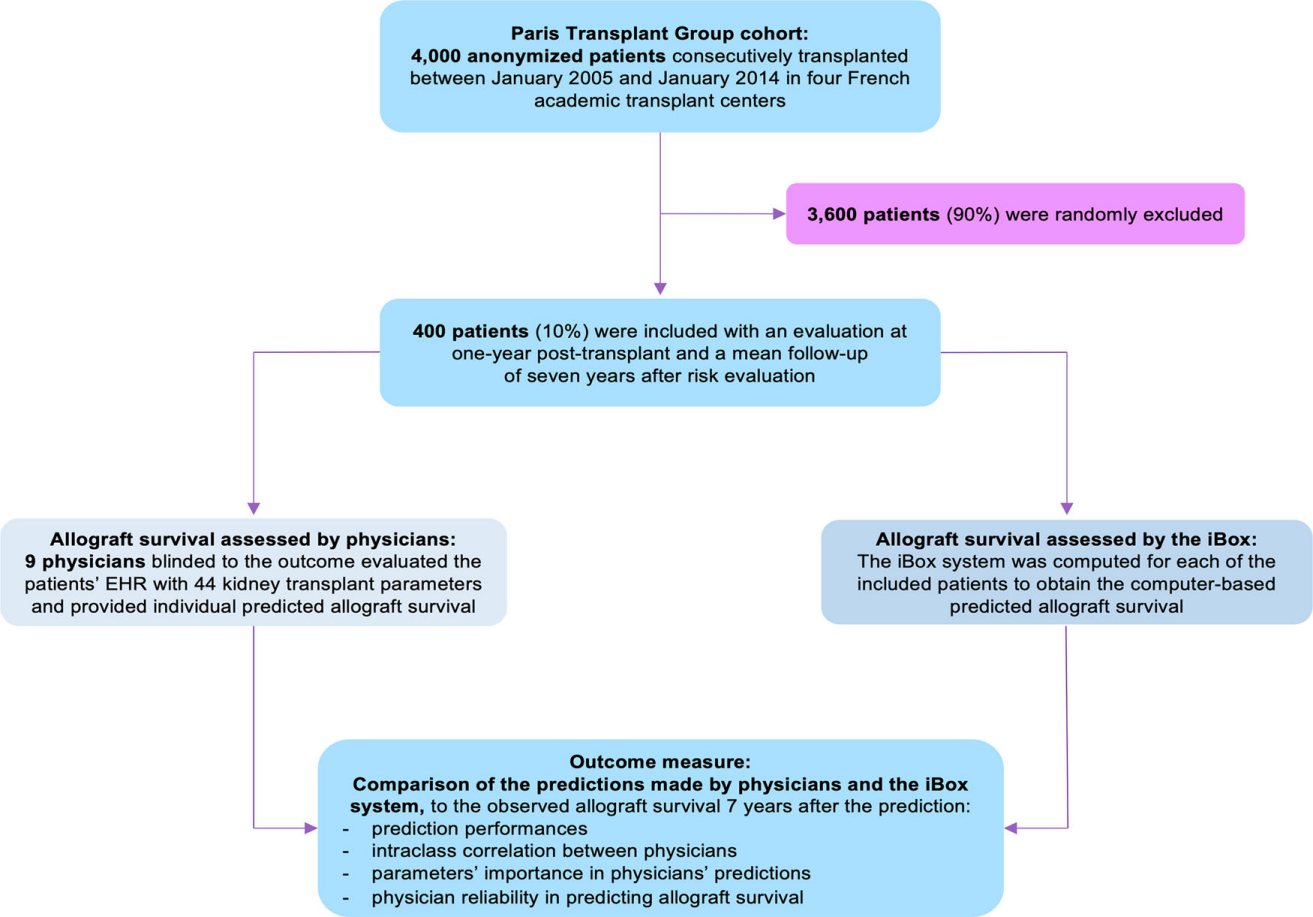
Study Flowchart. Four hundred patients were randomly selected from the Paris Transplant Group database, a multicenter prospective cohort involving 4000 patients consecutively transplanted between 2005 and 2014 from four French academic transplant centers (Necker and Saint-Louis hospitals, Paris; Foch hospital, Suresnes; Toulouse hospital, Toulouse). Forty-four parameters from the first year of transplantation were included and integrated into an anonymized Electronic Health Record (EHR) for evaluation by nine transplant physicians and the iBox prediction system. Allograft survival at seven years post-evaluation predicted by the physicians and the iBox were compared to the observed allograft survival to assess prediction performances. The agreement of physicians’ predictions was compared using intraclass correlation, and the most important parameters in physicians’ prediction were ranked and compared using mean decreased accuracy from a random forest algorithm and Fleiss kappa. EHR: electronic health record.

From the 4000 kidney transplant recipients of a qualified prospective multicentric cohort, 400 patients with an evaluation available at one-year post-transplant were randomly selected as detailed in the flow chart (Fig. 1). This cohort includes consecutive patients over 18 years of age prospectively enrolled at the time of kidney transplantation from a living or deceased donor at Necker Hospital, Saint-Louis Hospital, Foch Hospital, and Toulouse Hospital between 1 January 2005, and 1 January 2014, in France and has been previously reported and used to develop the iBox prognostication system^9^.

All data from this cohort were anonymized and prospectively entered at the time of transplantation, at the time of post-transplant allograft biopsies, and at each transplant anniversary by using a standardized protocol. The electronic case report form (eCRF), includes features routinely collected in health care and kidney transplant, comprised demographic characteristics (including recipients’ comorbidities, age, sex, height, and weight), transplant characteristics (including Donor type, Donor comorbidities, immunological risk defined by circulating anti-HLA Donor specific antibodies at time of transplantation), biological features (including glomerular filtration rate estimated (eGFR) by the Modification of Diet in Renal Disease Study equation^22^, urine protein/creatinine ratio^23^, and circulating anti-HLA donor-specific antibodies (DSA) specificities and concentrations), and allograft histologic data (including elementary lesion scores and diagnoses interpreted according to the Banff international classification for allograft pathology^24^). Data were retrieved from the database on March 2018 and allograft outcomes were prospectively assessed in the Paris Transplant Group cohort up to 1 January 2021. All patients provided written informed consent at the time of transplantation. The institutional review board of the Paris Transplant Group approved the protocol of the study (NCT03474003, IRB: #000119258). This database has been approved by the National French Commission for Bioinformatics, Data, and Patient Liberty: CNIL registration number: 363505.

### Patient anonymized electronic health record (EHR)

Patient risk evaluation, which was performed at one-year post-transplant according to the centers’ practices, comprised 44 transplant features included in the anonymized electronic health record (EHR). The features are detailed in Supplementary Table 1 and comprise: (i) Recipient characteristics including comorbidities, age, gender, number of years on dialysis, (ii) Donor characteristics including age, deceased/living, cause of death, history of hypertension or diabetes, (iii) Transplant characteristics including the number of HLA mismatches, cold ischemia time, induction therapy, delayed graft function, (iv) Biological features at time of evaluation including the kidney allograft function which was assessed by the glomerular filtration rate estimated (eGFR) by the Modification of Diet in Renal Disease Study equation^22^, and the proteinuria level using the urine protein/creatinine ratio^23^, (v) Immunological features at the time of transplant and evaluation, including circulating anti-HLA donor-specific antibodies against HLA-A, HLA-B, HLA-Cw, HLA-DR, HLA-DQ and HLA-DP which were assessed using single-antigen flow bead assays^25^ (mean fluorescence intensity and specificity), and (vi) Allograft pathology data according to the 2017 Banff international classification for allograft pathology^24^ (including elementary lesion scores and diagnoses).

### Enrollment of physicians

We recruited nine independent physicians to review the EHR of the 400 anonymized patients to predict the risk of long-term allograft failure. Physicians were included in the study if they met several criteria including: (i) no involvement in the iBox development study, (ii) active involvement in daily kidney transplant patient care, (iii) acceptance of a training tutorial to use the online anonymized EHR, (iv) consent to use the EHR blinded from the outcome to be compared to an algorithm, (v) accept to review each of the 400 anonymized EHR. We then stratified the physicians according to their clinical experience to approximate the landscape of daily transplant care: three were residents in nephrology or transplant surgery, three were fellows in a general nephrology unit or a kidney transplant unit, and three were assistant professors or full professors (seniors) in a kidney transplant unit. Details about each physician and their clinical experience are summarized in Supplementary Table 2.

### Outcome measures

The outcome of interest was the individual prediction performances assessed by transplant physicians and the AI system respectively to predict the risk of long-term allograft failure. Kidney allograft failure was defined as a patient’s definitive return to dialysis or pre-emptive kidney retransplantation. Patients who died with a functioning allograft were censored at the time of death as patients with a functional allograft.

### Artificial intelligence-based allograft failure prediction

Individual allograft survival probabilities of the 400 included patients were computed using the iBox algorithm^9^, a validated AI system designed to predict the risk of long-term allograft failure up to seven years after evaluation, as previously described^9^. For each patient, the iBox was calculated based on the β regression coefficients from the iBox study using 8 features available in the EHR, including baseline characteristics (time from transplant to evaluation), functional features (eGFR and protein/creatinine ratio), immunological features (MFI of the immunodominant circulating anti-HLA donor-specific antibodies), and histological features including microcirculation inflammation (g and ptc Banff scores), interstitial inflammation and tubulitis (i and t Banff scores), transplant glomerulopathy (cg Banff score), and interstitial fibrosis/tubular atrophy (IFTA Banff score). These 8 clinically meaningful features were found to be independently associated with allograft failure among 44 features commonly and routinely collected in kidney transplant centers. The iBox algorithm has been externally validated in randomized clinical trials and multiple independent cohorts in Europe, North and South America^9,26,27^.

### Physician-based allograft failure prediction

We created an online platform consisting of patient anonymized electronic health records (EHR) blinded to the allograft outcome and the iBox predictions. A visual of the online platform is shown in Supplementary Fig. 1.

The tasks of the physicians were as follows: (i) Read the EHR of each patient, (ii) Estimation of the long-term allograft survival at seven years after the time of evaluation, and selection of a percentage (0% = very high risk of allograft failure, 100% = very low risk of allograft failure), (iii) Selection of a score to further represent the risk of allograft failure from 0 (very high risk of allograft failure) to 10 (very low risk of allograft failure).

### Statistical analysis

Continuous variables were described using means and standard deviations (SDs) or medians and the interquartile ranges. We compared means and proportions between groups using Student’s *t*-test, analysis of variance (Mann–Whitney test for MFI) or the chi-square test (or Fisher’s exact test if appropriate). Allograft survival was estimated using the Kaplan-Meier method. The duration of follow-up started with the patient risk evaluation (starting point) up to the date of kidney allograft failure, or the end of follow-up (1 January 2021). For patients who died with a functioning allograft, allograft survival was censored at the time of death as a functional allograft.

#### Evaluation of the prediction performances

The accuracy of both the physicians’ predictions and the iBox prognostication system were assessed based on the discrimination using Harrell’s concordance index and with visual examination of calibration curves and median calibration error for each calibration plot (*rms* package in R). The median calibration error was assessed as the median of the absolute differences, for each quantile, between the predicted allograft failure, and the fraction of surviving allografts. Additionally, a decision curve analysis was performed to assess the clinical value using the net benefit of the iBox system compared with the physicians to predict allograft failure at seven years post-evaluation (*dcurves* package in R).

#### Evaluation of feature importance in the prediction

To identify the features driving each physician’s predictions, a random survival forest was performed for each physician. The mean decrease in accuracy was used to determine the relative importance of the first ten features that led to their predictions. We then ranked all features to obtain the overall top 10 features driving the physician’s judgment (*randomForestSRC* package in R). Fleiss kappa was used to measure inter-rater agreement between each physician’s ranking (*irr* package in R)^28^.

#### Evaluation of physician reliability to predict the risk of long-term allograft failure

The inter-rater reliability was investigated using intraclass correlation (ICC) to evaluate the physician’s agreement to predict each individual patient percentage of allograft survival at seven years post-evaluation. A two-way random effect for multiple raters/measurements ICC model was fit using Stata^29^.

To assess the intra-rater reliability, the relationship between the two predictions made by each physician (probabilities and risk scores of allograft failure) were compared using a linear regression model for each physician. In addition, the inter-rater reliability to score each individual patient was also tested using a two-way random effect for multiple raters/measurements ICC model in Stata^29^.

All analyses were performed using R (version 3.6.3, R Foundation for Statistical Computing) and Stata (StataCorp. 2017. Stata Statistical Software: Release 15. College Station, TX: StataCorp LP). Values of *p* < 0.05 were considered significant, and all tests were two-tailed.

### Reporting summary

Further information on research design is available in the Nature Research Reporting Summary linked to this article.

## Results

### Characteristics of the included patients at risk evaluation

The characteristics of the patients included at the time of transplantation are detailed in Supplementary Table 3 and were representative of the prospective multicentric cohort of 4000 kidney transplant recipients (see Supplementary Fig. 2). The mean patient age at the time of transplantation was 51.62 years (SD = 13.60), 224 were male (56.00%), and 341 allografts (85.25%) were from deceased donation. Regarding the immunological risk, 57 (14.25%) were previously transplanted, and 79 (19.75%) had circulating anti-HLA DSA at time of transplantation. At 7 years post-evaluation, 84 (21%) patients developed the primary outcome of allograft failure.

### Physicians and artificial intelligence-based prediction performances

**Fig. 2:**
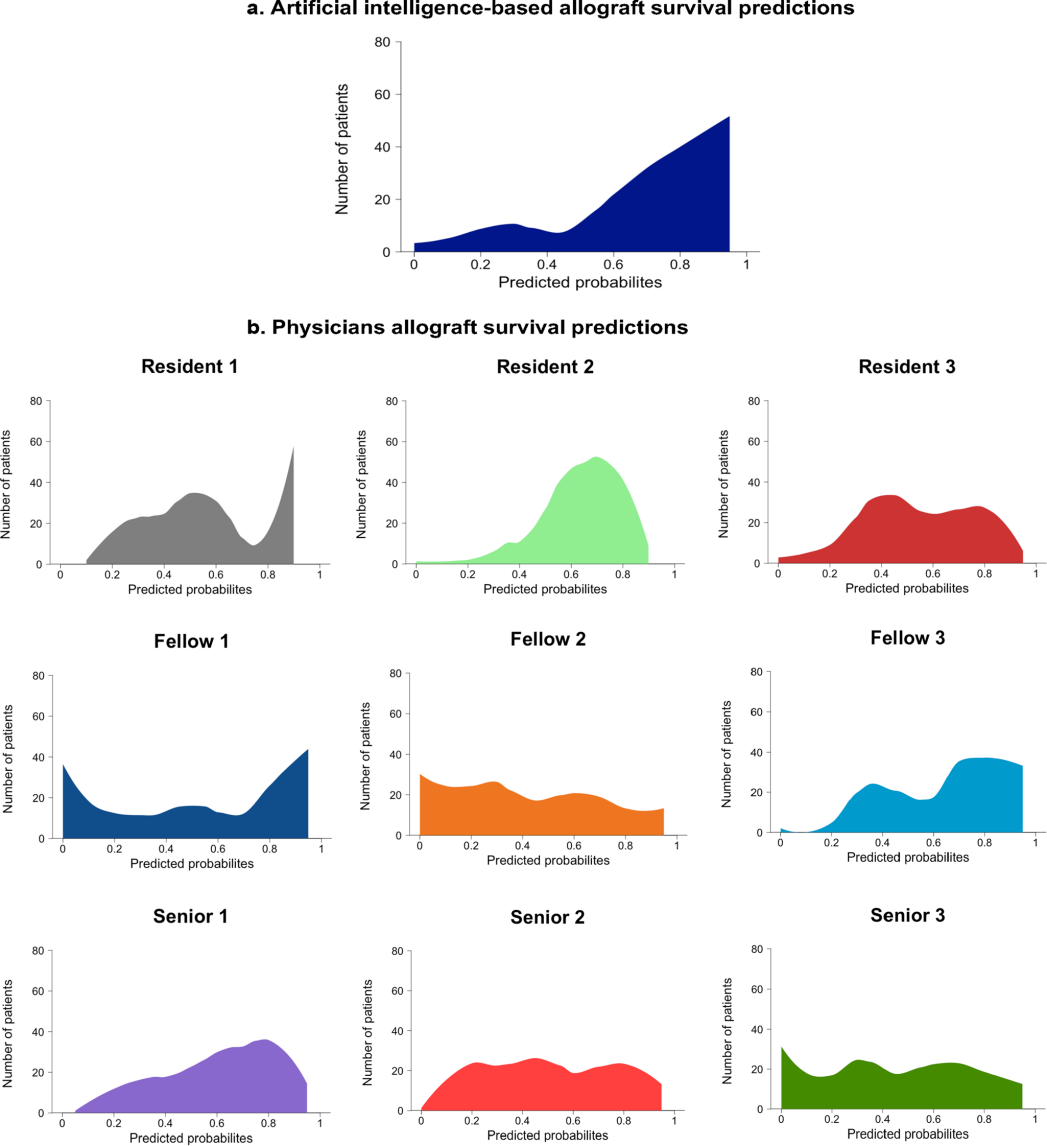
Distribution of predicted probabilities of allograft survival according: physicians vs artificial intelligence-based prediction system. *n* = 400 patients, nine transplant physicians and the iBox system. Density plot of the distribution of predicted probabilities. Each color corresponds to one physician or the iBox. The median and the interquartile range of the iBox prediction system (**a**) and each physician (**b**): iBox 78.3% [60.6–89.5]; Resident #1, 60% [40–80]; Resident #2, 70% [60–80], Resident #3, 57% [41–77], Fellow #1, 60% [25–90], Fellow #2, 40% [20–70], Fellow #3, 77% [50–82], Senior #1, 69.5% [50–82.5], Senior #2, 50% [30–80] and Senior #3, 47% [25.5–72.5].

The AI system (iBox) and physicians’ predicted probabilities had distinct distributions (*p* < 0.001, Fig. 2). The medians [IQR] of the kidney allograft survival probabilities were 78% [61–89] for the iBox, 60% [40–80] for Resident #1, 70% [60–80] for Resident #2, 57% [41–77] for Resident #3, 60% [25–90] for Fellow #1, 40% [20–70] for Fellow #2, 77% [50–82] for Fellow #3, 69.5% [50–82] for Senior #1, 50% [30–80] for Senior #2 and 47% [25–72] for Senior #3 (Fig. 2). The kidney allograft survival prediction distribution of the subgroups of physicians with the same clinical experience were also significantly different (*p* < 0.001 for Residents, *p* < 0.001 for Fellows and *p* < 0.001 for Seniors physicians).

The discrimination at 7 years post-evaluation was higher for the iBox algorithm with a discrimination of 0.789 (Supplementary Fig. 3b), while the physicians had a lower and heterogenous discrimination performances with 0.638 for Resident #1, 0.754 for Resident #2, 0.755 for Resident #3, 0.771 for Fellow #1, 0.786 for Fellow #2, 0.736 for Fellow #3, 0.763 for Senior #1, 0.767 for Senior #2, 0.703 for Senior #3.

**Fig. 3:**
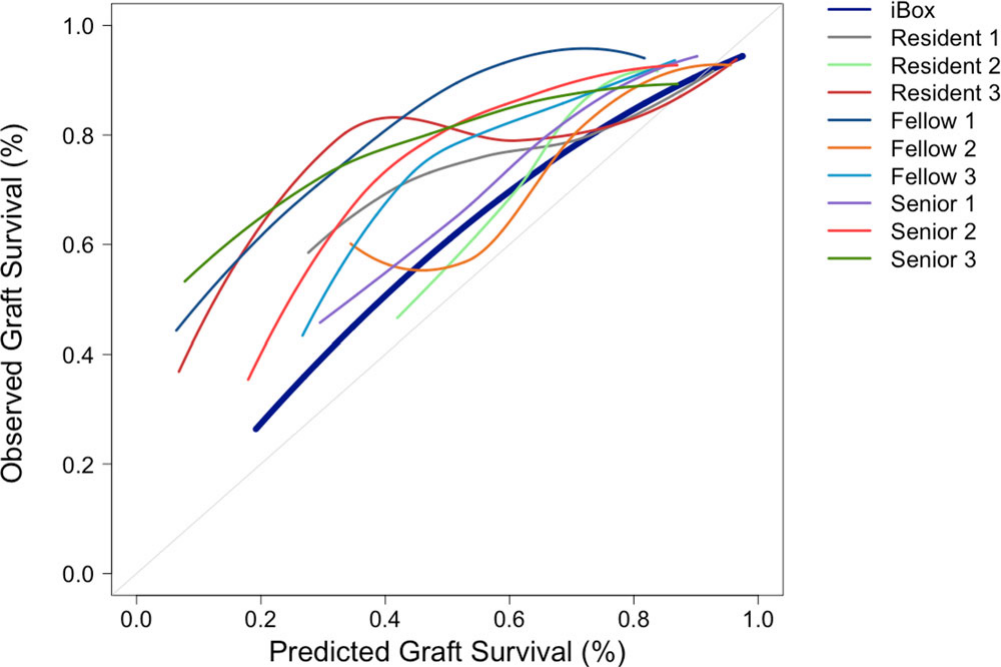
Smoothed calibration curve comparing the observed graft survival and the predicted graft survival of each physicians’ and the artificial intelligence-based prediction system. *n* = 400 patients, nine transplant physicians and the iBox system. Calibration plots at seven years post risk evaluation for the four hundred patients. Vertical axis is observed proportion of grafts surviving at seven years. Average predicted probability (predicted survival; *x*-axis) was plotted against Kaplan–Meier estimate (observed overall survival; *y*-axis). Gray line represents perfectly calibrated model. Except the dark blue line which represents the smoothed iBox predictions, each plot represents Physicians’ predictions. The intercept and slope were 0.135 and 0.87 for the iBox system, 0.48 and 0.46 for Resident #1, 0.00 and 1.14 for Resident #2, 0.31 and 0.78 for Resident #3, 0.47 and 0.51 for Fellow #1, 0.48 and 0.66 for Fellow #2, 0.32 and 0.65 for Fellow #3, 0.22 and 0.83 for Senior #1, 0.33 and 0.79 for Senior #2, 0.55 and 0.44 for Senior #3.

The calibration plots showed that the AI system predictions were more reliable. On average, physicians tended to overestimate the risk of graft failure at risk evaluation, regardless of the level of experience (Fig. 3). The percentage of median calibration errors (Supplementary Fig. 3a) was low for the iBox algorithm with 5.79% IQR [4.40–7.72] confirming an adequate calibration, while physicians had a higher percentage of, respectively, 18.10% IQR [7.33–29.68] for Resident #1, 9.60% IQR [7.05–10.66] for Resident #2, 19.16% IQR [15.65–24.36] for Resident #3, 19.44% IQR [5.35–29.24] for Fellow #1, 35.87% IQR [35.36–39.44] for Fellow #2, 8.62% IQR [2.92–9.31] for Fellow #3, 12.64% IQR [12.29–13.67] for Senior #1, 23.83% IQR[16.8–29.44] for Senior #2, 33.03% IQR [20.58–43.98] for Senior #3.

Decision curve analysis showed that the iBox has greater net benefit across a range of thresholds higher than 2% compared with most physicians (*n* = 8/9, 88.9%) and 10% for all physicians (Supplementary Fig. 4). At a threshold of 20% (percentage of allograft failure observed), the net benefit of the iBox system is that the model identified 46 more cases per 1000 without increasing the number of patients treated unnecessarily. Conversely, the net benefit was lower for all the physicians.

### Agreement between physicians to predict the risk of long-term allograft failure

We used intraclass correlation (ICC) to compare a quantitative variable with multiple raters. The individual graft survival probabilities predicted by physicians showed wide heterogeneity with a moderate intraclass correlation of 0.58 95% CI [0.51–0.64] between all physicians. We also compared the intraclass correlation by considering the clinical experience and achievement of each physician. The inter-rater reliability remains poor with an ICC of 0.48 95% CI [0.39–0.56] for Residents and moderate for Fellows and Seniors physicians with an ICC of 0.61 95% CI [0.39–0.74] and 0.59 95% CI [0.45–0.69] respectively.

### Evaluation of the features driving the predictions made by the physicians

**Fig. 4:**
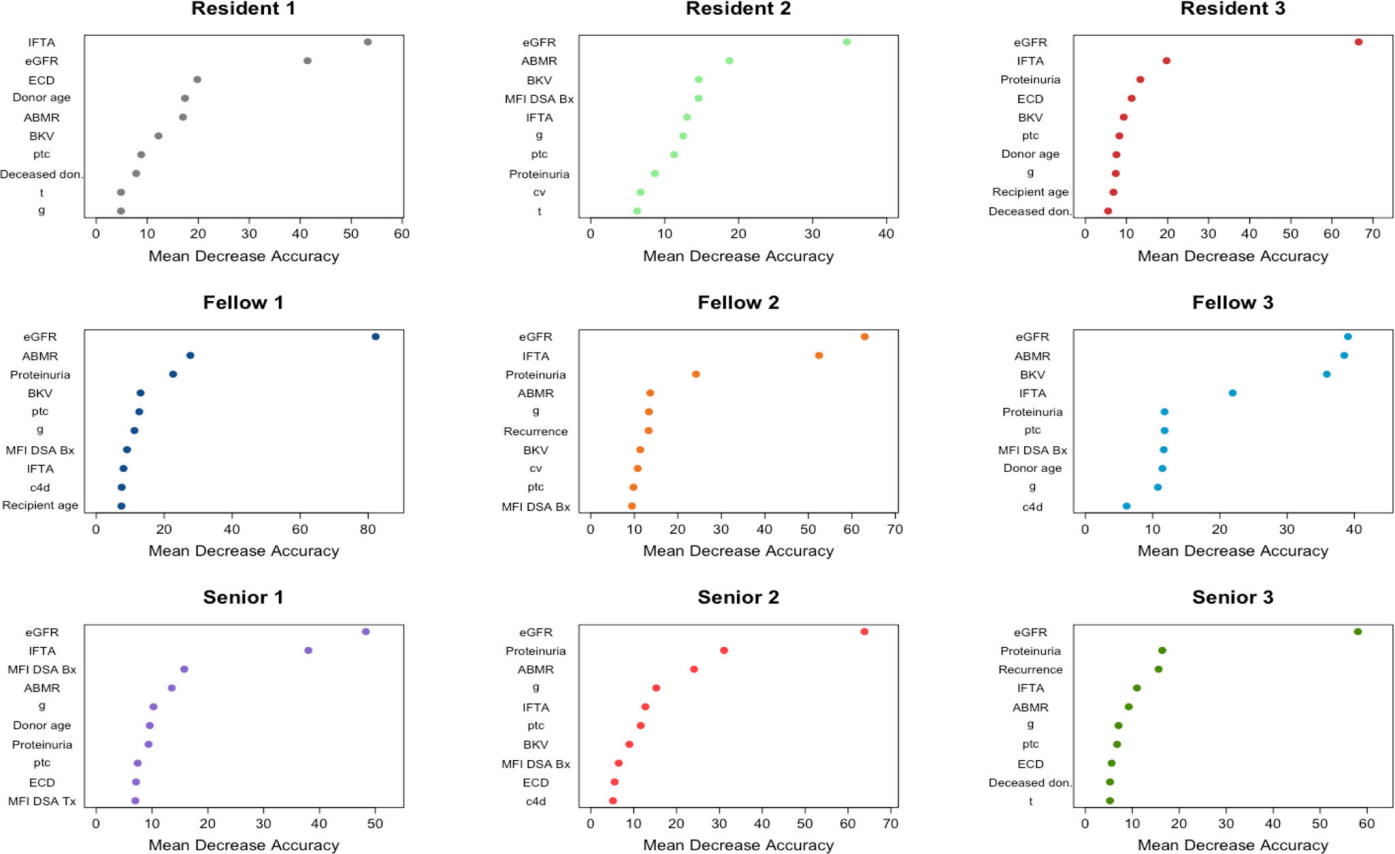
Ranking of the ten most important features driving the physicians’ predictions. *n* =  400 patients and nine transplant physicians. We ranked the features importance for each physician based on the mean decrease accuracy from each physicians’ Random Survival Forest. Each plot expresses how much accuracy is lost in the prediction by excluding each parameter. The features are presented from descending importance. The higher the value of mean decrease accuracy, the higher the importance of the feature in the model. The cg and i Banff scores were not found in any physicians; the t Banff score was found in 3 (33%) physicians and the MFI of the immunodominant anti-HLA DSA was found in 6 (66%) physicians. ABMR antibody mediated rejection, i interstitial inflammation Banff score, t tubulitis Banff score, g glomerulitis Banff score, ptc peritubular capillaritis Banff score, cv arterial intimal fibrosis Banff score, ah arteriolar hyalinosis Banff score, c4d c4d staining of peritubular capillaritis Banff score, IFTA interstitial fibrosis and tubular atrophy, MFI mean fluoresence intensity, DSA donor specific antibody, ECD expanded criteria donor, eGFR estimated Glomerular Filtration Rate.

The most consistent feature between the physicians and the iBox system was eGFR with a Fleiss kappa of 0.75 (*p* < 0.001). The hierarchy of the features were broadly different across physicians with an overall Fleiss kappa of 0.13 (Supplementary Table 4). Several features constituting the iBox system, hence independently associated with the risk of long-term allograft failure, were not driving forces in the physicians’ prognostic judgment (Fig. 4). Indeed, apart from the eGFR, the other features present in the iBox score with a slight agreement between all physicians were the histological Banff scores of glomerulonephritis [g score] (Fleiss kappa 0.17) and peritubular capillaritis [ptc score] (Fleiss kappa 0.11). The circulating anti-HLA donor specific antibody status at time of the evaluation was not ranked by a third of the physicians (two residents and one senior, Fig. 4). We then stratified the agreement by their clinical experience. The agreement remained poor with a Fleiss kappa of 0.02 for residents, 0.20 for fellows and 0.10 for seniors (Supplementary Table 4). Among physicians with the same clinical experience, the features with the best agreement were expanded criteria donor (Fleiss kappa 0.46) and eGFR (Fleiss kappa 0.26) for residents, eGFR (Fleiss kappa 1.00) and donor age (Fleiss kappa 0.46) for fellows and eGFR (Fleiss kappa 1.00) only for seniors.

### Evaluation of physician reliability to predict the risk of long-term allograft failure

To assess physician reliability between estimating the percentage of allograft survival and scoring a risk of allograft failure at seven years post-evaluation, a linear regression was performed for each physician (Supplementary Fig. 5). A strong negative linear relationship corresponding to a high reliability between the two assessments was found for each physician with a R-squared of 0.905 for Resident #1 (*p* < 0.001), 0.766 for Resident #2 (*p* < 0.001), 0.860 for Resident #3 (*p* < 0.001), 0.985 for Fellow #1 (*p* < 0.001), 0.929 for Fellow #2 (*p* < 0.001), 0.973 for Fellow #3 (*p* < 0.001), 0.992 for Senior #1 (*p* < 0.001), 0.934 for Senior #2 (*p* < 0.0001) and 0.822 for Senior #3 (*p* < 0.001).

We also investigated the intraclass correlation of physicians to score the risk of allograft failure. The intraclass correlation (ICC) remained moderate at 0.55% CI [0.46–0.62] between all physicians, when taking into account the achievement of each physician, the results were consistent with an ICC of 0.52 95% CI [0.19–0.71] for Residents, 0.47 95% CI [0.41–0.53] for Fellows and 0.57 95% CI [0.42–0.68] for Seniors.

## Discussion

In this study, we investigated the prediction performances of an AI system and 9 transplant physicians with distinct clinical experiences, in assessing the risk of long-term allograft failure after kidney transplant. We showed that the iBox had better prediction performances than physicians, regardless of their experience. We also showed that physicians had limited performance, reproducibility, and consistency to predict the risk of long-term allograft failure.

Interestingly, few physicians had a discrimination close to the iBox but they all tended to overestimate the risk of allograft failure while the iBox showed a good discrimination and a strong, stable calibration. The predicted risk corresponds to the actual outcome for a large combination of predictor values. In addition, the predicted probabilities of long-term allograft failure were highly heterogeneous between physician estimates, while the iBox is stable.

We further supported this argument by ranking the feature importance for both the iBox and physicians. The physicians demonstrated a high heterogeneity in the choice of features that best predict the risk of long-term allograft failure. This result was not influenced by clinical experience, underscoring the possibility that this heterogeneity may be present in all physicians regardless of their experience. Overall, we demonstrated that physicians estimated that some key features independently associated with allograft failure described in the literature and included in the iBox were not, in their professional opinion, the most relevant driving forces^6,30,31^.

Therefore, as one given patient has one given risk of losing the allograft according to a spectrum of parameters, this disparity demonstrates that even if one physician may sometimes accurately predict the risk of a patient, other physicians are unlikely to have the same accuracy. This can lead to heterogeneity of practices for the same patient between physicians with potential invasive examinations or unnecessary treatments without benefit to the patient. Better predicting kidney allograft survival can help physicians improve risk stratification with reinforced surveillance for patients at high risk.

Overall, these findings illustrate that the iBox can inform physicians’ prognostic judgment and therefore decision-making and monitoring. As such, the iBox is a promising companion tool in daily transplant practice.

Kidney transplantation is a health care field representative of the quest for precision medicine over the past two decades^32^. Transplant physicians are overwhelmed with increasing data that are subject to many changes in definition and evaluation. For instance, the international Banff classification of allograft pathology has been updated every two years since 1991, making the interpretation of histological lesions increasingly complex^33,34^. Furthermore, to detect anti-HLA donor-specific antibodies, the Luminex single antigen bead assay technique is used worldwide, but remains difficult to interpret for physicians due to the use of different cut-offs and interpretations between laboratories^25,35^. Together, these ongoing changes represent a diverse knowledge that requires, for a physician, a long experience in transplant care and research to be correctly understood and integrated. Therefore in this context, the iBox, which suffers from less bias associated with memory and computation capability than humans, is likely to be of valuable assistance in transplant care.

More generally, this study reinforces the effort already made by researchers to compare machines to humans in the diagnosis or prognosis based on clinical data. This effort has often been focused on how machines could outperform physicians for image classification and disease diagnosis, but also more recently in patient prognostication of short-term outcomes^36–39^. However, to the best of our knowledge, this study is one of the first to compare long-term outcome predictions from a validated integrative prognostication system to physician predictions using EHR.

Despite its superior prediction performances, the iBox system will not replace physicians. The value of the iBox is its integration of a large spectrum of parameters from miscellaneous sources highly associated with the risk of long-term allograft failure in kidney transplantation^9^. However, it does not integrate the complexity of the physician-patient relationship, which involves many subtleties that contribute to decision-making. Further, additional specific data such as complications related to immunosuppressive treatment and events like cancer and infections have an important influence on clinical decisions. They are however not considered by the iBox system, although it may indirectly integrate the consequences of these events. Therefore, even though physicians predict with lower accuracy the risk of long-term allograft failure, they also have a large overview of the patient that cannot be currently reached by the machine. Thus, instead of opposing these two perspectives, the iBox should be considered as a companion tool that helps the physician in the evaluation of the patient, and thereby may serve as a support decision-making tool.

## Conclusion

In conclusion, the present study demonstrates the overall limited performance of physicians to accurately predict individual risk of long-term allograft outcome compared with a validated AI prognostic system. This study also shows the potential added value of the iBox prognostication system to inform physicians in their prognostic judgment in kidney transplantation, supporting the use of computer assistance to help physicians in the decision-making process.

## Supplementary information

Supplementary Information

Reporting Summary

## Data Availability

All source data to reproduce the main Figs. 2–4 and the Supplementary Figs. 3–5 are deposited into the synapse public repository^40^. Additional data to reproduce Supplementary Fig. 2 are available upon reasonable request. Technical appendix is available from the corresponding author at alexandre.loupy@inserm.fr. Study protocol is available on clinicaltrials.gov: NCT04918199.

## Code availability

The code to reproduce the analyses of the main Figs. 2–4 is deposited into the synapse public repository^40^.
