# Supplementary material for: Comparison of artificial intelligence and human-based prediction and stratification of the risk of long-term kidney allograft failure
Source: Commun Med (Lond). 2022 Nov 23;2:150. doi: 10.1038/s43856-022-00201-9 (PMC9684574; doi:10.1038/s43856-022-00201-9)

Supplemental Material

Supplementary Table 1: Features prospectively collected and assessed in the anonymized electronic health record

Features	Labels
Recipient features	
**Recipient id**	
**Recipient Age at transplantation**	Unit in years
**Gender**	Male or Female
**BMI**	in Kg/m^2
**Nephropathy (End Stage Kidney Disease)**	Glomerular diseases
	Tubulointerstitial diseases
	Vascular diseases
	Cystic and congenital diseases
	Other
**Dialysis before transplant**	No or Yes
**Retransplantation**	No or Yes
Donor features	
**Age**	Unit in years
**Gender**	Male or Female
**Donor type**	deceased or living
**ECD if DD (Deceased Donor)**	No or Yes
**BMI**	in Kg/m^2
**Hypertension**	No or Yes
**Diabetes**	No or Yes
**Creatinine at baseline**	Unit in µmol/l
**eGFR at baseline**	Unit in $ml/min/1.73m^2$
**Cerebro-vascular death**	No or Yes
Baseline_features	
**Cold ischemia time**	Unit in hours
**Delayed graft function**	No or Yes
**Mismatches HLA (A, B, DR)**	
**Induction Therapy**	ATG
	Basiliximab
	Other
**Day zero DSA**	No or Yes
**Class Day zero DSA**	1 or 2
**MFI Day zero DSA**	
Biopsy_features	
**Screening biopsy**	No or Yes
**Time post-transplant**	In months
Banff lesion grading system	
**i**	**0** (< 10 %), **1** (10-25%), **2** (26-50%), **3** (> 50%)
**t**	**0** (No), **1** (1-4), **2** (5-10), **3** (> 10)
**v**	**0** (No), **1** (Mild), **2** (Severe), **3** (Transmural)
**g**	**0** (No), **1** (< 25%), **2** (25-75%), **3** (> 75%)
**ptc**	**0** (< 10% or > 3), **1** (> 10 % + 3 to 4 Leucocytes), **2** (> 10 % + 5 to 10 Leucocytes), **3** (> 10 % + > 10 [illegible]
**cg**	**0** (No), **1** (1-25%), **2** (26-50%), **3** (> 50%)
**ah**	**0** (No), **1** (Mild), **2** (Moderate), **3** (Severe)
**cv**	**0** (No), **1** (< 25%), **2** (26-50%), **3** (> 50%)
**ci**	**0** (< 5%), **1** (6-25%), **2** (26-50%), **3** (> 50%)
**ct**	**0** (No), **1** (< 25%), **2** (26-50%), **3** (> 50%)
**C4d**	**0, 1, 2, 3**
Banff Diagnoses	
**ABMR**	No or Yes
**TCMR**	No or Yes
**Borderline**	No or Yes
**Recurrence**	No or Yes
**BK virus Nephropathy**	No or Yes
**Thrombotic microangiopathy**	No or Yes
**CNI Toxicity**	No or Yes

Supplementary Table 2: clinical experience and position of each physician

<table>
<tr><th>Physicians</th><th>Position</th><th>Speciality</th><th>Years of clinical experience</th></tr>
<tr><td rowspan="3">Residents</td><td>Resident</td><td>Nephrologist candidate</td><td>1 year before end of residency</td></tr>
<tr><td>Resident</td><td>Nephrologist candidate</td><td>4 years before end of residency</td></tr>
<tr><td>Resident</td><td>Transplant surgeon</td><td>1 Year after residency</td></tr>
<tr><td rowspan="3">Fellows</td><td>Fellow</td><td>MD in nephrology</td><td>4 years after residency</td></tr>
<tr><td>Fellow</td><td>MD in nephrology</td><td>1 year after residency</td></tr>
<tr><td>Fellow</td><td>MD in nephrology</td><td>Two years after residency</td></tr>
<tr><td rowspan="3">Seniors</td><td>Transplant nephrologist,
Assistant Professor</td><td>MD in nephrology</td><td>8 years after residency</td></tr>
<tr><td>Transplant nephrologist
Head of the kidney transplant unit</td><td>MD in nephrology</td><td>12 years after residency</td></tr>
<tr><td>Transplant nephrologist
Assistant Professor</td><td>MD in nephrology</td><td>7 years after residency</td></tr>
</table>

Supplementary Table 3: Patients characteristics

	n	Patients (n=400)
**Recipient demographics**		
**Age** (years), mean (SD)	400	51.62 (13.60)
**Gender male**, No. (%)	400	224 (56.0)
**Body Mass Index, mean (SD)**	383	24.36 (4.43)
**End-stage kidney disease causes**	400	
**Glomerulonephritis,** No. (%)		106 (26.50)
**Diabetes**, No. (%)		46 (11.50)
**Vascular**, No. (%)		27 (6.75)
**Other,** No. (%)		221 (55.25)
**Transplant characteristics**		
**Donor age** (years), mean (SD)	400	54.95 (16.23)
**Donor male gender,** No. (%)	400	208 (52.0)
**Donor hypertension,** No. (%)	391	136 (34.78)
**Donor diabetes mellitus,** No. (%)	386	32 (8.29)
**Donor serum creatinine >1.5 mg/dL,** No. (%)	397	46 (11.59)
**Donor type**		
**Deceased donor**, No. (%)	400	341 (85.25)
**Death from cerebrovascular disease,** No. (%)	341	211 (61.88)
**Expanded criteria donor,** No. (%)	400	185 (46.25)
**Prior kidney transplant,** No. (%)	400	57 (14.25)
**Cold ischemia time** (hours), mean (SD)	397	16.77 (8.94)
**Delayed graft function†,** No. (%)	390	125 (32.05)
**HLA-A/B/DR mismatch**, mean (SD), number	400	3.88 (1.28)
**Anti-HLA DSA at time of transplant,** No. (%)	400	79 (19.75)
**Class of the immunodominant DSA at time of transplant**	79	
**I, No. (%)**		36 (45.6)
**II, No. (%)**		43 (54.4)

Abbreviations: HLA: human leucocyte antigen; DSA: donor specific antibody; ABMR: antibody mediated rejection; TCMR: T-cell mediated rejection.

† Delayed graft function was defined as the use of dialysis in the first postoperative week.

Supplementary Table 4: classification of features by level of agreement using Fleiss Kappa

	Overall<br>n=9	Resident<br>n=3	Fellow<br>n=3	Senior<br>n=3
**Overall Fleiss Kappa**	**0.13**	**0.02**	**0.2**	**0.1**
**classification of features by level of agreement**				
**Poor<br><0**	• Proteinuria,<br>• ABMR,<br>• i, ah, c4d Banff scores<br>• Recurrence<br>• MFI of the DSA at time of transplant	• Proteinuria,<br>• ABMR,<br>• cv, g, IFTA Banff scores<br>• BK virus nephropathy<br>• MFI of the DSA at time of transplant and at risk evaluation<br>• Donor age<br>• Deceased donor<br>• Recipient age	• ABMR,<br>• cv, c4d Banff scores<br>• BK virus nephropathy<br>• MFI of the DSA at risk evaluation<br>• ECD donor status<br>• Recurrence<br>• Deceased donor<br>• Recipient age	• ABMR,<br>• g, i, ah, IFTA Banff scores<br>• BK virus nephropathy<br>• MFI of the DSA at time of transplant and at risk evaluation<br>• ECD donor status<br>• Recurrence<br>• Donor age<br>• Recipient age<br>• Deceased donor
**Slight<br>0.01 – 0.2**	• g*, ptc*, cv Banff scores<br>• Recipient age<br>• IFTA,<br>• MFI of the DSA at risk evaluation<br>• BK virus nephropathy<br>• ECD donor status<br>• Donor age			
**Fair<br>0.21 – 0.4**		• eGFR*,<br>• ptc Banff score	• IFTA,<br>• g, ptc Banff scores<br>• Proteinuria	• ptc Banff scores<br>• Proteinuria
**Moderate<br>0.41 – 0.6**		• ECD donor status*	• Donor age*	
**Substantial<br>0.61 – 0.8**	• eGFR*			
**Almost perfect<br>0.81 – 1**			• eGFR*	• eGFR*

n = 400 patients and 9 transplant physicians.

ABMR: antibody mediated rejection; i: interstitial inflammation Banff score; t: tubulitis Banff score; g: gomerulitis Banff score; ptc: peritubular capillaritis Banff score; cv: arterial intimal fibrosis Banff score; ah: arteriolar hyalinosis Banff score; c4d: c4d staining of peritubular capillaritis Banff score; IFTA: interstitial fibrosis and tubular atrophy; MFI: mean fluoresence intensity; DSA: Donor Specific Antibody; ECD: Expanded Criteria Donor; eGFR: estimated Glomerular Filtration Rate.

*features with an agreement p-value <0.05

Supplementary Figure 1: Visual of the Anonymized Electronic Health Record

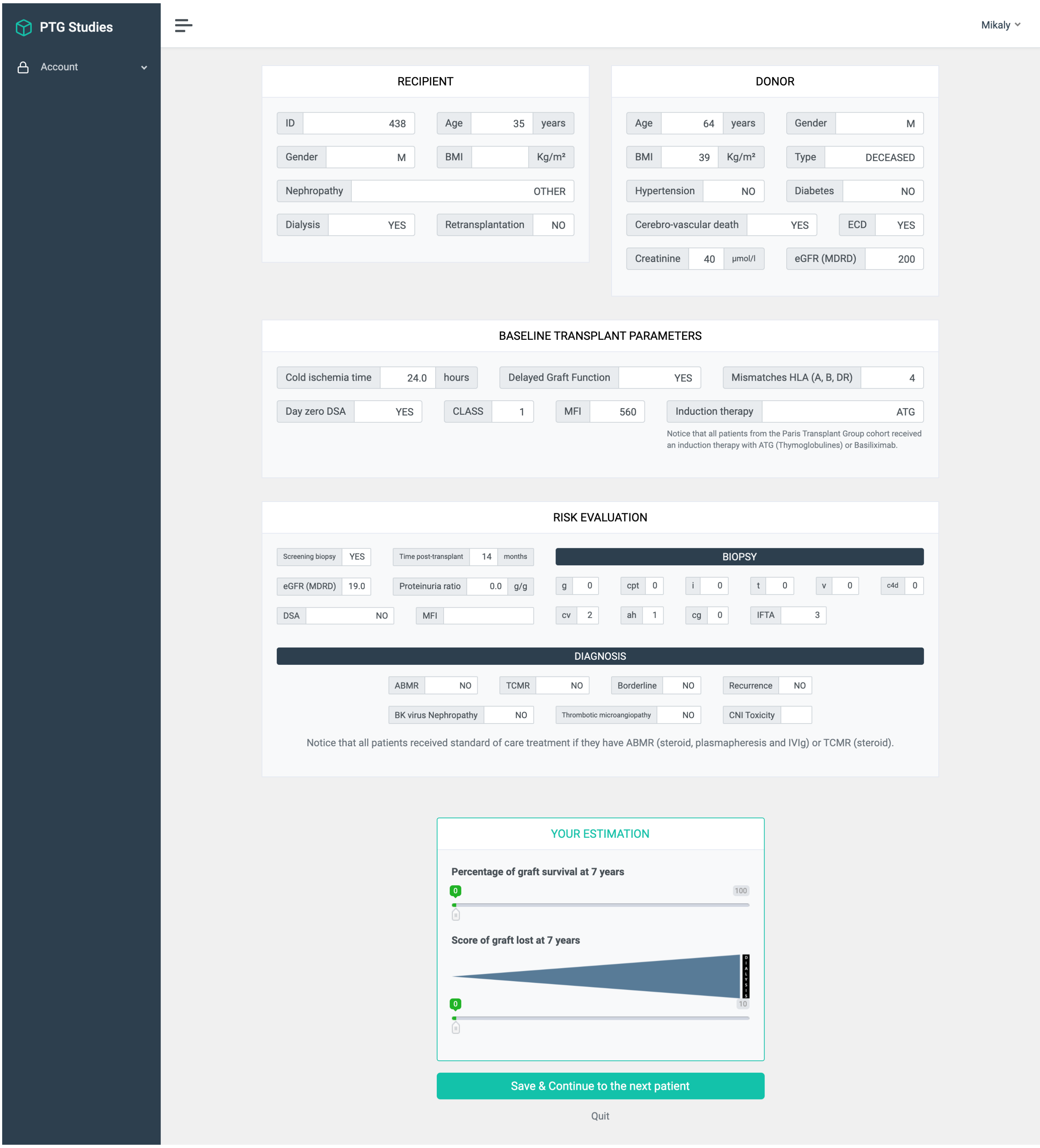

Supplementary figure 2: Comparison of the baseline characteristics between the Paris Transplant group cohort and the subset of 400 patients.

n = 4000 patients.The twelve most relevant features were compared between the Paris Transplant group cohort (n=4,000 patients) and the subset of 400 patients. Continuous features were described using boxplot and compared using Student's t-test (Recipient age, Cold ischaemia time and HLA mismatch A, B, DR). All boxplots are drawn from first quartile to third quartile, with a line at the median. Whiskers indicate 5th and 95th percentile absolute error. Categorical features were described using barplot and compared using chi-square test (Recipient gender, End stage kidney disease cause, Donor gender, Donor type, Donor history of diabetes mellitus, Donor with serum creatinine >1.5 mg/dL, Prior kidney transplantation, Delayed Graft function and Anti-HLA Donor specific antibody at time of transplant).

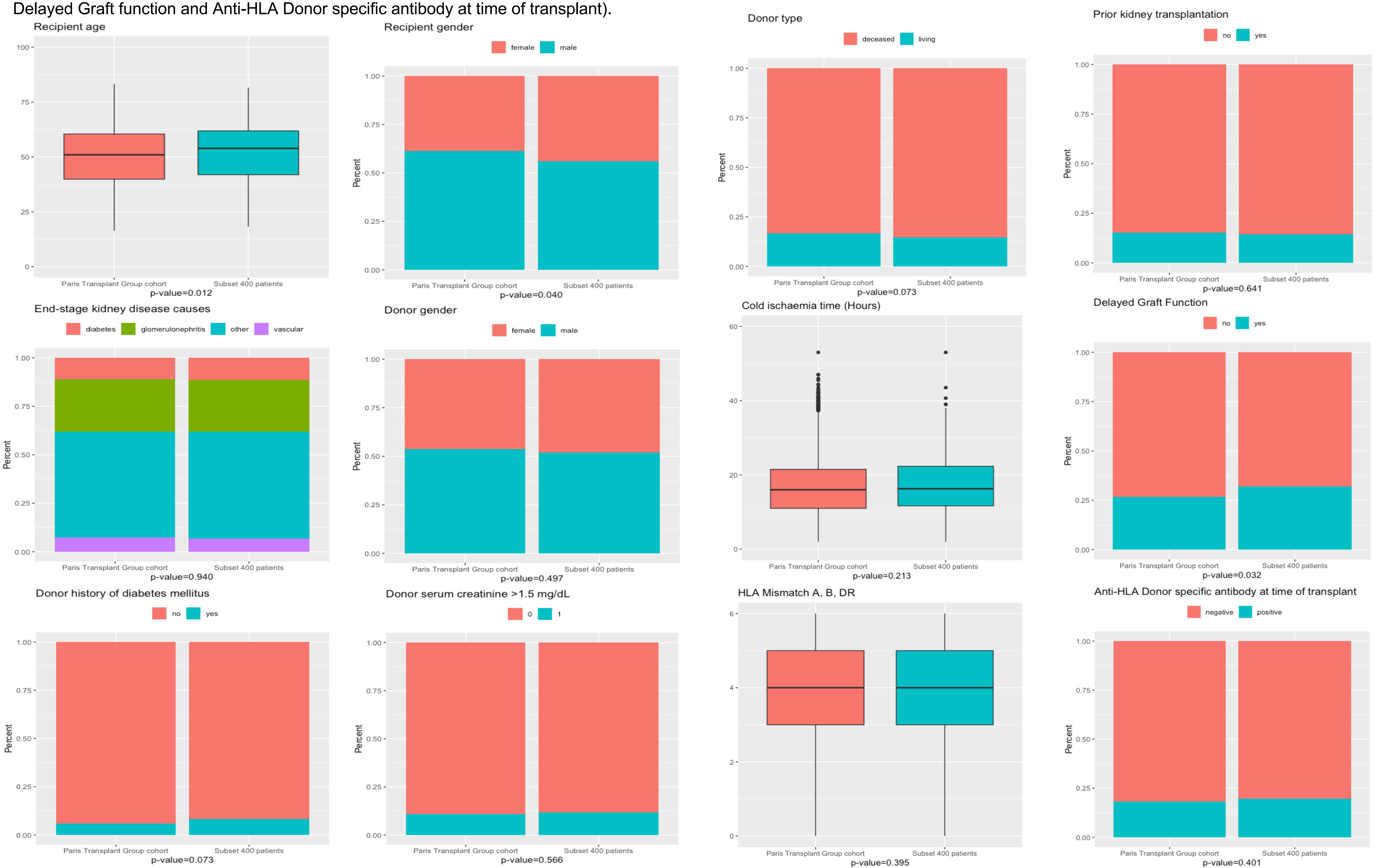

Supplementary Figure 3: percentage of median calibration errors and discrimination of each physician and the iBox system at 7 years post-evaluation

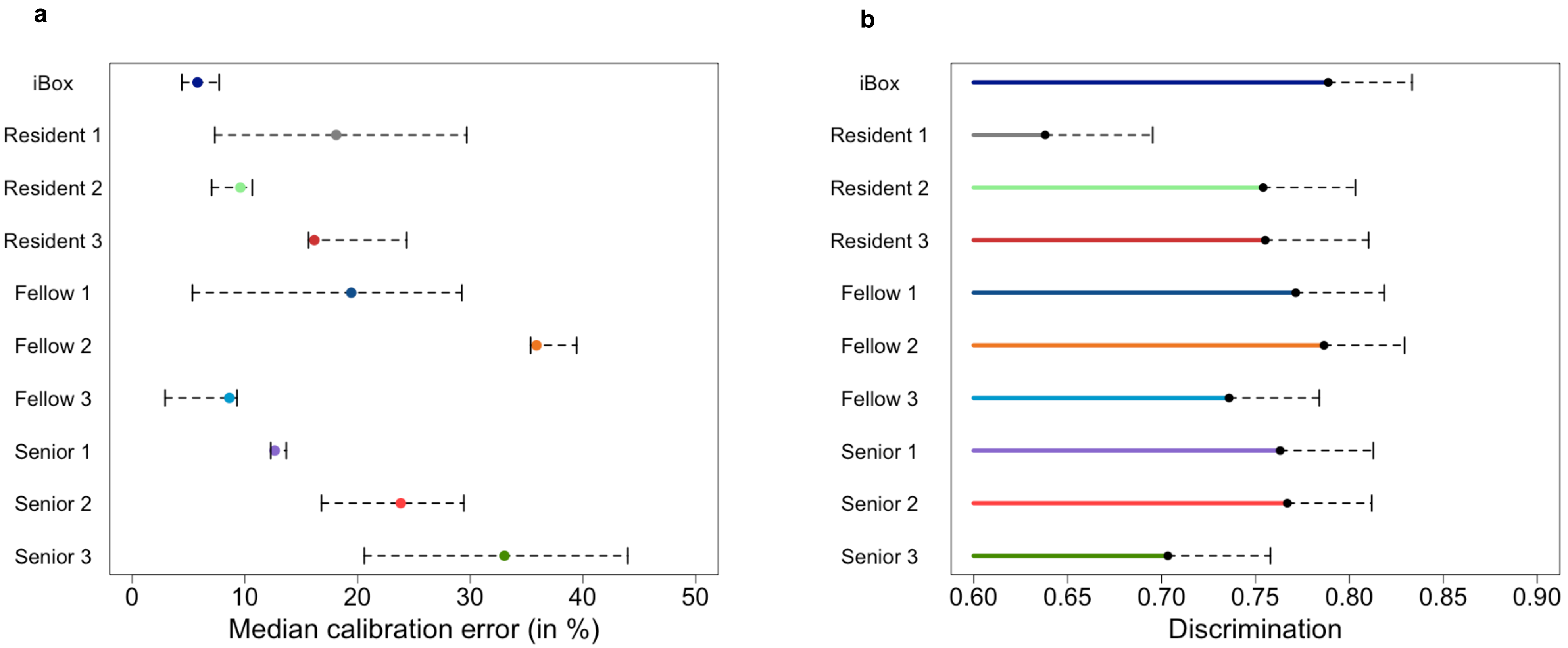

n = 400 patients, 9 transplant physicians and the iBox system. a) Percentage of median calibration error of each calibration curve, each color represents each physician and the iBox. The percentage of median calibration error was respectively, 5.79% IQR[4.4-7.72] for the iBox, 18.1% IQR[7.33-29.68] for Resident #1, 9.6% IQR[7.05-10.66] for Resident #2, 19.16% IQR[15.65-24.36] for Resident #3, 19.44% IQR[5.35-29.24] for Fellow #1, 35.87% IQR[35.36-39.44] for Fellow #2, 8.62% IQR[2.92-9.31] for Fellow #3, 12.64% IQR[12.29-13.67] for Senior #1, 23.83% IQR[16.8-29.44] for Senior #2, 33.03% IQR[20.58-43.98] for Senior #3. Each dot represents the median and error bars indicate the interquartile range.

b) Discrimination (Harrell's concordance index) of each physician and the iBox at 7 years post-evaluation, 0.789 95%CI [0.744-0.833] for the iBox, 0.638 95%CI [0.581-0.695]for Resident #1, 0.754 95%CI [0.705-0.803] for Resident #2, 0.755 95%CI [0.700-0.810] for Resident #3, 0.771 95%CI [0.724-0.818] for Fellow #1, 0.786 95%CI [0.744-0.829] for Fellow #2, 0.736 f95%CI [0.688-0.784] or Fellow #3, 0.763 95%CI [0.714-0.813]for Senior #1, 0.767 95%CI [0.722-0.812] for Senior #2 and 0.703 95%CI [0.649-0.758] for Senior #3, respectively. Each dot represents the discrimination and error bars indicate the upper bounds of the 95% confidence interval.

Supplementary Figure 4: Decision curve analysis between prediction of the iBox system and each physician at 7 years post-evaluation.

n = 400 patients, 9 transplant physicians and the iBox system. The x-axis indicates the threshold probability for the outcome of graft failure. The y-axis indicates the net benefit. Two extreme strategies were added as references, the Black line represents the net benefit of treating all patients at risk of allograft failure and the light grey line represents the net Benefit of consider all patients at low risk of allograft failure. The iBox system and all physicians net benefit are plotted.

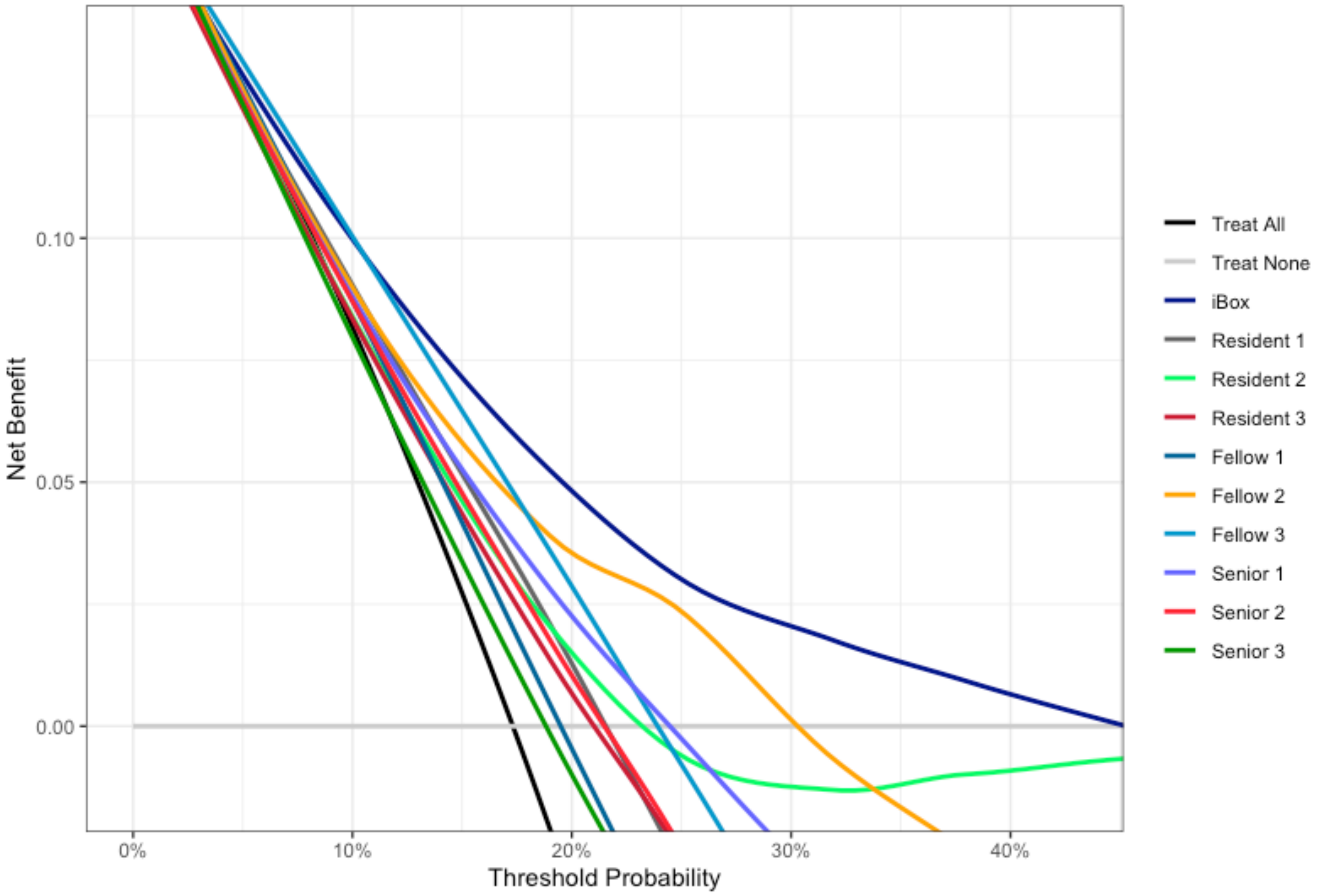

Supplementary Figure 5: linear regression between prediction of allograft survival and score of allograft failure for each physician.
n = 400 patients and 9 transplant physicians. R-squared of 0.905 for Resident #1 (p<0.001), 0.766 for Resident #2 (p<0.001), 0.860 for Resident #3 (p<0.001), 0.985 for Fellow #1 (p<0.001), 0.929 for Fellow #2 (p<0.001), 0.973 for Fellow #3 (p<0.001), 0.992 for Senior #1 (p<0.001), 0.934 for Senior #2 (p<0.0001) and 0.822 for Senior #3 (p<0.001).

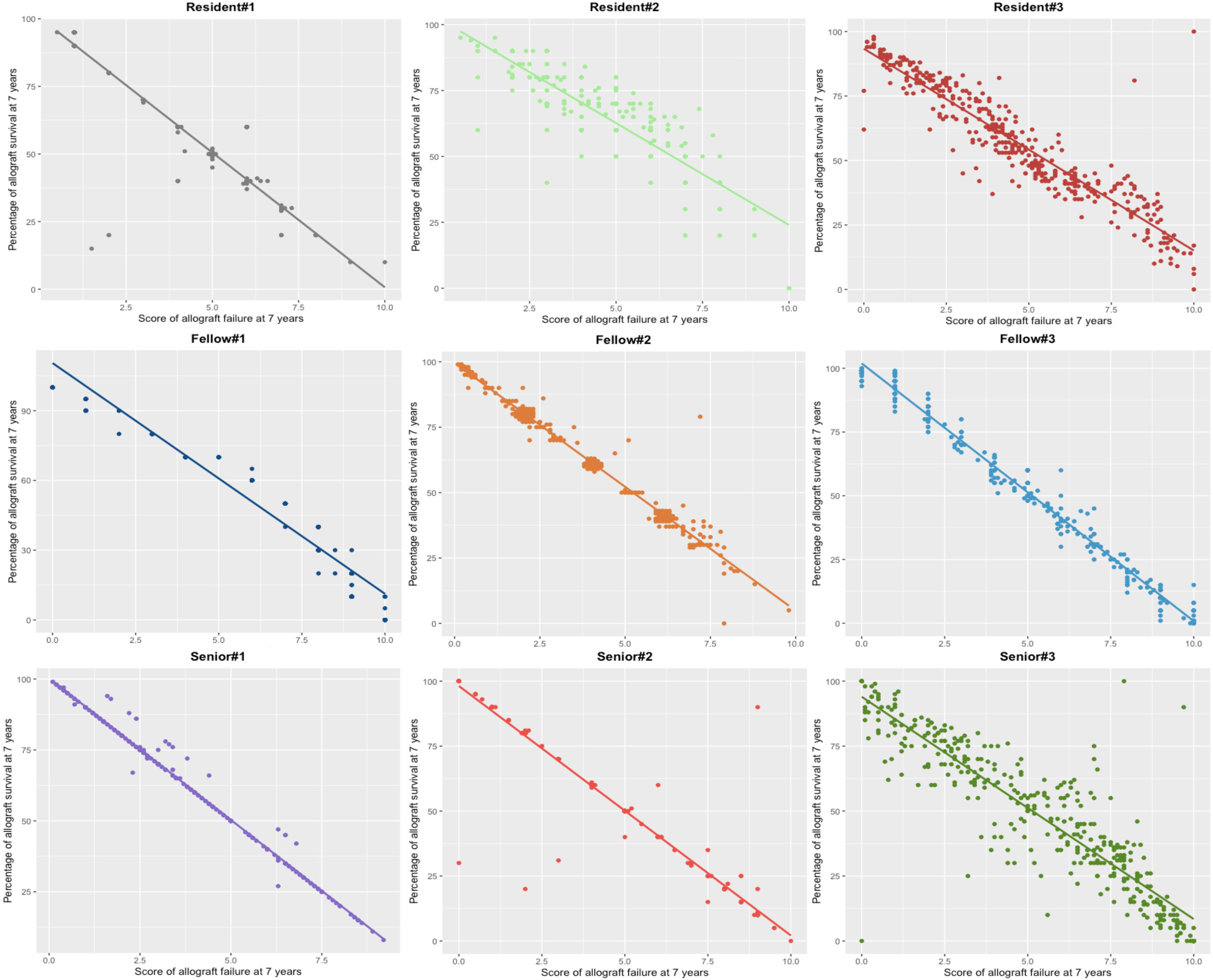

Supplement: Supplementary file 1 — Supplementary Information [file 43856_2022_201_MOESM1_ESM.pdf]
