# Supplementary material for: Comparison of artificial intelligence and human-based prediction and stratification of the risk of long-term kidney allograft failure
Source: Commun Med (Lond). 2022 Nov 23;2:150. doi: 10.1038/s43856-022-00201-9 (PMC9684574; doi:10.1038/s43856-022-00201-9)
Supplement: Supplementary file 2 — Reporting Summary [file 43856_2022_201_MOESM2_ESM.pdf]

nature research

Corresponding author(s): Alexandre Loupy
Last updated by author(s): Oct 12, 2022

Reporting Summary

Nature Research wishes to improve the reproducibility of the work that we publish. This form provides structure for consistency and transparency in reporting. For further information on Nature Research policies, see our Editorial Policies and the Editorial Policy Checklist.

Statistics

For all statistical analyses, confirm that the following items are present in the figure legend, table legend, main text, or Methods section.

n/a	Confirmed	
☐	☒	The exact sample size (*n*) for each experimental group/condition, given as a discrete number and unit of measurement
☐	☒	A statement on whether measurements were taken from distinct samples or whether the same sample was measured repeatedly
☐	☒	The statistical test(s) used AND whether they are one- or two-sided<br>*Only common tests should be described solely by name; describe more complex techniques in the Methods section.*
☐	☒	A description of all covariates tested
☐	☒	A description of any assumptions or corrections, such as tests of normality and adjustment for multiple comparisons
☐	☒	A full description of the statistical parameters including central tendency (e.g. means) or other basic estimates (e.g. regression coefficient) AND variation (e.g. standard deviation) or associated estimates of uncertainty (e.g. confidence intervals)
☐	☒	For null hypothesis testing, the test statistic (e.g. *F*, *t*, *r*) with confidence intervals, effect sizes, degrees of freedom and *P* value noted<br>*Give P values as exact values whenever suitable.*
☒	☐	For Bayesian analysis, information on the choice of priors and Markov chain Monte Carlo settings
☐	☒	For hierarchical and complex designs, identification of the appropriate level for tests and full reporting of outcomes
☐	☒	Estimates of effect sizes (e.g. Cohen's *d*, Pearson's *r*), indicating how they were calculated

Our web collection on statistics for biologists contains articles on many of the points above.

Software and code

Policy information about availability of computer code

Data collection	No software were used
Data analysis	R (version 3.6.3, R Foundation for Statistical Computing) and STATA (StataCorp. 2017. Stata Statistical Software: Release 15. College Station, TX: StataCorp LP). rms & randomForestSRC & irr packages in R.

For manuscripts utilizing custom algorithms or software that are central to the research but not yet described in published literature, software must be made available to editors and reviewers. We strongly encourage code deposition in a community repository (e.g. GitHub). See the Nature Research guidelines for submitting code & software for further information.

Data

Policy information about availability of data

All manuscripts must include a data availability statement. This statement should provide the following information, where applicable:

- Accession codes, unique identifiers, or web links for publicly available datasets
- A list of figures that have associated raw data
- A description of any restrictions on data availability

All source data to reproduce the main figures and the supplementary figure 3-5 are deposited into the synapse public repository: 10.7303/syn26958595. Additional data to reproduce supplementary figure 2 are available upon reasonable request. Technical appendix is available from the corresponding author at alexandre.loupy@inserm.fr. Study protocol is available on clinicaltrials.gov: NCT04918199.

Field-specific reporting

Please select the one below that is the best fit for your research. If you are not sure, read the appropriate sections before making your selection.

☒ Life sciences ☐ Behavioural & social sciences ☐ Ecological, evolutionary & environmental sciences

For a reference copy of the document with all sections, see nature.com/documents/nr-reporting-summary-flat.pdf

Life sciences study design

All studies must disclose on these points even when the disclosure is negative.

Sample size	400 patients were randomnly selected in the Paris Transplant Group cohort of 4,000 patients.
Data exclusions	iBox not computable, patient without risk evaluation at one year post-transplant
Replication	All the analyses we performed and reproduce by the 2 first coauthors
Randomization	Not relevant
Blinding	All physicians were blinded from the patient outcome at time of risk evaluation

Reporting for specific materials, systems and methods

We require information from authors about some types of materials, experimental systems and methods used in many studies. Here, indicate whether each material, system or method listed is relevant to your study. If you are not sure if a list item applies to your research, read the appropriate section before selecting a response.

Materials & experimental systems

n/a	Involved in the study	
☒	☐	Antibodies
☒	☐	Eukaryotic cell lines
☒	☐	Palaeontology and archaeology
☒	☐	Animals and other organisms
☐	☒	Human research participants
☐	☒	Clinical data
☒	☐	Dual use research of concern

Methods

n/a	Involved in the study	
☒	☐	ChIP-seq
☒	☐	Flow cytometry
☒	☐	MRI-based neuroimaging

Human research participants

Policy information about studies involving human research participants

Population characteristics	4,000 kidney transplant recipients from a qualified prospective multicentric cohort. This cohort gathers consecutive patients over 18 years of age prospectively enrolled at the time of kidney transplantation from a living or deceased donor at Necker Hospital, Saint-Louis Hospital, Foch Hospital, and Toulouse Hospital between January 1, 2005, and January 1, 2014, in France. All data from this cohort were anonymized and prospectively entered at the time of transplantation, at the time of post-transplant allograft biopsies, and at each transplant anniversary by using a standardized protocol. The electronic case report form (eCRF) including features routinely collected in kidney transplant comprised demographic characteristics (including recipients' comorbidities, age, sex, and transplant characteristics), biological features (including kidney allograft function, proteinuria, and circulating anti-HLA antibody specificities and concentrations), and allograft histologic data (including elementary lesion scores and diagnoses). Data were retrieved from the database on March 2018
Recruitment	From the 4,000 kidney transplant recipients of a qualified prospective multicentric cohort, 400 patients were randomly included with an evaluation available at one-year post-transplant. This cohort gathers consecutive patients over 18 years of age prospectively enrolled at the time of kidney transplantation from a living or deceased donor at Necker Hospital, Saint-Louis Hospital, Foch Hospital, and Toulouse Hospital between January 1, 2005, and January 1, 2014, in France. See flow chart figure 1.
Ethics oversight	All patients provided written informed consent at the time of transplantation. The institutional review board of the Paris Transplant Group approved the protocol of the study (NCT03474003, IRB: #000119258). This database has been approved by the the National French Commission for Bioinformatics, Data, and Patient Liberty: CNIL registration number: 363505.

Note that full information on the approval of the study protocol must also be provided in the manuscript.

Clinical data

Policy information about clinical studies

All manuscripts should comply with the ICMJE guidelines for publication of clinical research and a completed CONSORT checklist must be included with all submissions.

Clinical trial registration	NCT04918199
Study protocol	Protocol previously published : Loupy A, Aubert O, Orandi BJ, et al. Prediction system for risk of allograft loss in patients receiving kidney transplants: international derivation and validation study. BMJ 2019;366:l4923. Prospective multicentric cohort gathering consecutive patients over 18 years of age prospectively enrolled at the time of kidney transplantation from a living or deceased donor at Necker Hospital, Saint-Louis Hospital, Foch Hospital, and Toulouse Hospital between January 1, 2005, and January 1, 2014, in France. All data from this cohort were anonymized and prospectively entered at the time of transplantation, at the time of post-transplant allograft biopsies, and at each transplant anniversary by using a standardized protocol. The electronic case report form (eCRF) including features routinely collected in kidney transplant comprised demographic characteristics (including recipients' comorbidities, age, sex, and transplant characteristics), biological features (including kidney allograft function, proteinuria, and circulating anti-HLA antibody specificities and concentrations), and allograft histologic data (including elementary lesion scores and diagnoses). Data were retrieved from the database on March 2018
Data collection	All data from this cohort were anonymized and prospectively entered at the time of transplantation, at the time of post-transplant allograft biopsies, and at each transplant anniversary by using a standardized protocol.
Outcomes	The outcome of interest was the individual prediction performances assessed by transplant physician and the computer-based system respectively to predict the risk of long-term allograft failure. Kidney allograft failure was defined as a patient's definitive return to dialysis or pre-emptive kidney retransplantation. Patients who died with a functioning allograft were censored at the time of death as patients with a functional allograft. Outcomes were prospectively assessed in the Paris Transplant Group cohort up to January 1st, 2021.
